# Nanocellulose coated paper diagnostic to measure glucose concentration in human blood

**DOI:** 10.3389/fbioe.2022.1052242

**Published:** 2022-11-22

**Authors:** Laila Hossain, Marisa De Francesco, Patricia Tedja, Joanne Tanner, Gil Garnier

**Affiliations:** Department of Chemical and Biological Engineering, Bioresource Processing Research Institute of Australia (BioPRIA), Monash University, Clayton, VIC, Australia

**Keywords:** nanocellulose, paper, enzyme, glucose oxidase, glucose sensor, diagnostic, blood

## Abstract

A new generation of rapid, easy to use and robust colorimetric point of care (POC) nanocellulose coated-paper sensors to measure glucose concentration in blood is presented in this study. The cellulose gel containing the enzyme with co-additive is coated and dried onto a paper substrate. Nanocellulose gel is used to store, immobilize and stabilize enzymes within its structure to prolong enzyme function and enhance its availability. Here, we immobilize glucose oxidase within the gel structure to produce a simple colorimetric blood glucose sensor. Increase in blood glucose concentration increases the concentration of reaction product which decreases the system pH detected by the pH indicative dye entrapped in the nanocellulose gel. The sensor produces a color change from red to orange as pH decreases due to the enzymatic reaction of glucose into gluconic acid and hydrogen peroxide. This sensor can measure glucose concentrations of 7–13 mM (medical range for diabetes control) at temperatures of 4°C–40°C. Stability tests confirm that no denaturation of enzyme occurs by measuring enzyme activity after 4 weeks. A prototype device is designed to instantly measure the glucose concentration from blood in a two steps process: 1) red blood cell separation and 2) quantification of glucose by color change. This study demonstrates nanocellulose sensor as an economical, robust, and sensitive diagnostic technology platform for a broad spectrum of diseases.

## Graphical Abstract

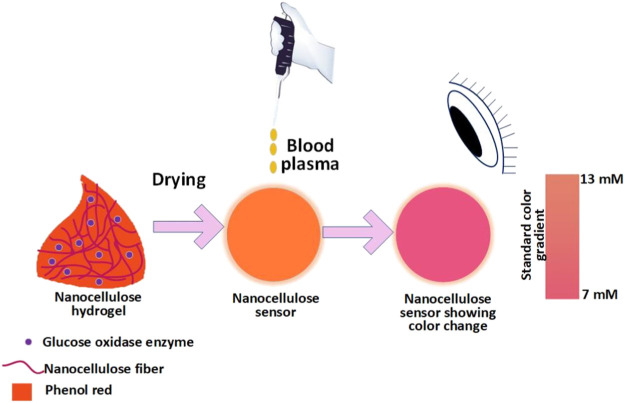

## 1 Introduction

Blood glucose measurement is important as high blood glucose levels are linked to adverse health outcomes in patients with diseases such as diabetics mellitus (Basiruddin and Swain, 2016), which can cause serious damage to the kidneys, blood vessels and heart over time. The number of people with diabetes worldwide is expected to double from 171 million in 2000 to 366 million by 2030 (Wild et al., 2004) with developing countries being the most affected (Hossain et al., 2007). According to the World Health Organization, around 1.5 million deaths were directly related to diabetes in 2019 (WHO, 2021). The rate of deaths associated with diabetes increases with remoteness, socioeconomic disadvantage, and ethnicity. In 2018, casualties were twice as high for remote or very remote communities compared to major cities, and four times as high for Aboriginal and Torres Strait Islanders compared to non-Indigenous Australians (Australian Institute of Health and Welfare, 2020).

There are three main types of diabetes: Type 1, Type 2 and gestational. Type 1 diabetes is an autoimmune disease which results in the inability of the body to produce insulin; it is more common in younger people. Type 2 diabetes develops when the body becomes resistant to the effect of insulin or the body’s ability to produce insulin gradually decreases over time; it usually develops in middle age and older people (Isley and Molitch, 2005; Ndisang et al., 2017). Gestational diabetes is a form of diabetes that occurs during pregnancy, which generally resolves following birth. However, women who develop gestational diabetes have an increased risk of developing type 2 diabetes later in life (Australia, 2021). More than 95% of patients having diabetes are type 2 (Kharroubi and Darwish, 2015). Diabetes is diagnosed as having a blood sugar level exceeding 7 mM after fasting or exceeding 11.1 mM 2 h after eating (World Health and International Diabetes, 2006). Prediabetes is defined as a blood sugar level exceeding 6.1 mM after fasting or 7.8 mM 2 h after eating (Alberti and Zimmet, 1998).

Continuous blood glucose monitoring for diabetic people is important to maintain health. Glucose concentration is the highest in the arterial circulation. However, lab tests are usually performed using venous blood samples. There are a few types of blood tests performed in pathology depending on the patient’s conditions which includes glycated hemoglobin (HbA1c), fasting, 2 h after having meal, glucose tolerance test and random blood glucose test. Blood glucose level is measured 2 h after having a meal to test how the body’s early respond to digestion. Blood glucose level increases sharply after a meal; however, the pancreas releases insulin to help assimilate the sugar from the blood into the cells of muscles and other tissues. Within 2 h of a meal, the glucose level in blood should return to normal. Random blood glucose test is performed if diabetes is suspected, and fasting blood glucose can confirm the diagnosis. HbA1c level is measured for diabetes patients to determine the recent average blood glucose level. It is also helpful to determine prediabetes as it provides the average glucose level for the past two to three months.

There are three basic approaches for measuring blood glucose in the laboratory: the reducing, the condensation and the enzymatic method. The reducing method is the oldest where the blood sample is treated with an alkaline copper solution to produce an insoluble cuprous oxide. This cuprous oxide is reacted with phosphomolybdate to form a molybdenum blue color complex which is read by spectrophotometer at 420 nm. This method was abandoned in the clinical laboratory as it is time consuming and includes the fructose concentration in the blood, which can yield erroneous results. In the condensation approach, O-toluidine reacts with glucose in blood to produce the green colored glucosamine. The intensity of this color complex is also measured by spectrophotometer to detect the glucose level. However, O-toluidine is highly corrosive and toxic, so this approach was also phased out from the laboratory (McMilin, 1990). The most common method in pathology or laboratory is to centrifuge the whole blood sample to collect plasma and test it using a semi-automatic or automatic biochemistry system with an enzyme which generates some colorimetric complex of intensity proportional to the glucose concentration (Nguyen, 2018). However, most of these lab-based tests are not suitable for home use.

A glucometer is very common to determine glucose level at home from whole blood. Glucose concentration in blood plasma is usually 10%–15% higher than in whole blood. Most laboratories/pathologies measure glucose level in plasma, so there are many glucometers which provide a “plasma equivalent” to compare users results with lab test (Rajaratnam and Pathmanathan, 2011). In the most common glucometer, the embedded enzyme (glucose oxidase) on the electrode induces an electric current through the strip which is proportional to the glucose amount (Kotwal and Pandit, 2012; Ansari et al., 2021). These types of conductivity-based biosensors are robust and accurate, but tend to be expensive, and therefore not suitable for a global application of diabetes diagnostics.

A direct paper-based testing device would provide a convenient alternative. Hydrogel and superabsorbent networks provide good support media as the sensing or detection locus where enzyme reacts with glucose to form some detectable complex. Several studies investigated glucose concentration by measuring the swelling or deswelling of hydrogels (Lin et al., 2010; Guenther et al., 2011). Glucose concentration was also determined by fluorescence quenching. Quantum dots (QDs) and carbon dots (CDs) are commonly used for fluorescent labelled sensor to detect glucose. However, reading blood sugar by fluorescent quenching is not feasible for home use. Glucose concentration in sweat, blood and saliva was monitored by the contact angle of analytes on a pH responsive surface or fluorescent nanohybrid (Gao et al., 2018; Cui et al., 2020). There are also optical sensors which provide different spectra based on glucose concentration and pH (Song et al., 2006; Khan et al., 2017; Lin et al., 2018; Wang et al., 2020). However, spectrum analysis at home is not a feasible option. Dilutions or pre-treatment of human blood is another disadvantage of most of these methods. Lee et al. (2019) recently reported colorimetric detection of glucose from human blood without pre-treatment or dilution. However, a post treatment is required for color intensity determination by eye. Further, aqueous sensors typically limit stability for long term storage and are more difficult to use. Another recent study developed nanocellulose based biosensor to determine glucose concentration; however, the sensor color change with glucose concentration was optimized for urine sample (Neubauerova et al., 2020).

**FIGURE 1:**
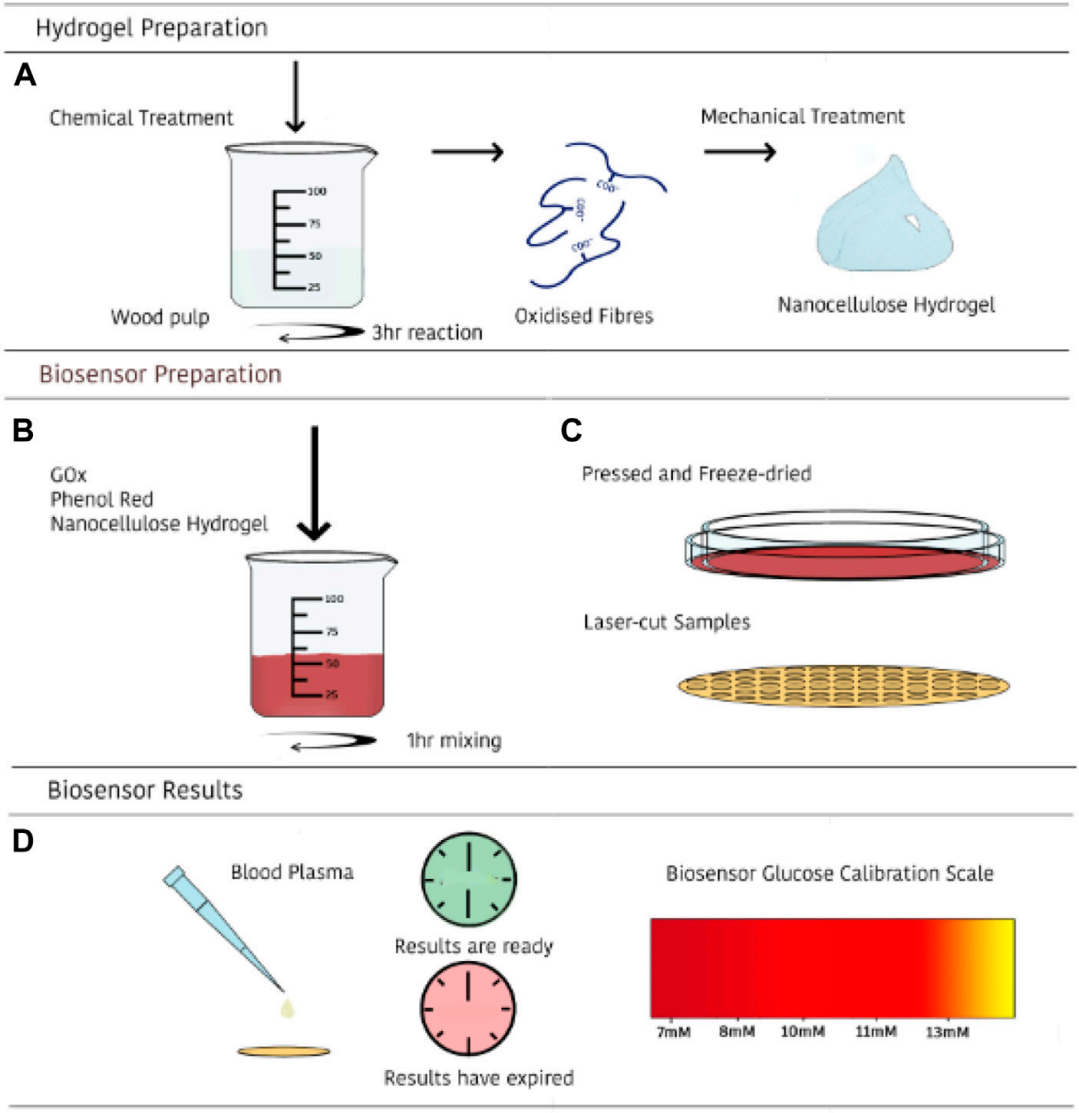
Schematic illustration of the preparation and use of the nanocellulose sensing diagnostic for measuring glucose concentration in human blood. First, a nanocellulose hydrogel of controlled COOH content and rheology is prepared **(A)**; second, a selected enzyme is immobilized onto the nanocellulose hydrogel **(B)**; third, nanocellulose hydrogel is freeze dried into a nanofoam with immobilized enzyme (adsorbed and entrapped), and converted into a diagnostic **(C)**; last, blood plasma is directly introduced—or processed by filtration of RBC- and glucose content displayed by color change **(D)**.

Here, we explore nanocellulose hydrogel as enzyme enhancer and stabilizer for paper-based diagnostic device. The aqueous and porous structure of nanocellulose hydrogel (Raghuwanshi and Garnier, 2019a; Raghuwanshi and Garnier, 2019b) combined with their exceptional biocompatibility (Curvello et al., 2019a; Curvello et al., 2021a; Curvello et al., 2021b) makes them attractive media for stabilizing and immobilizing fragile biomolecules such as enzymes (Curvello et al., 2019b). Enzyme immobilized hydrogels are freeze-dried into smart nanocellulose foams/superabsorbent with sensing ability. The colorimetric technique selected for the detection of diabetes from blood plasma is a pH-based reaction. This technique combines glucose oxidase and phenol red to induce a color change from red to orange as decreases pH in the foam (Figure 1). After quantifying the sensor ability to measure blood glucose, a device is designed to quantify glucose level from whole blood utilizing microfluidics. Blood is passed thought a paper layer with wicking kinetics engineered for optimized reaction time. Red blood cells (RBC) are filtered *via* a membrane in the next layer, allowing blood plasma to transport to the sensing zone where the enzymatic catalytic reaction decreases pH, directly shown by a color change. This study focuses on the sensor for reproducibility and accuracy. Nanocellulose sensors are tested for a blood plasma glucose ranging from 7 to 13 mM for diabetes control. A protocol to measure sensitivity and stability at different temperatures is presented to develop robust nanocellulose sensor for POC device. Last, shelf life is measured over 4 weeks for providing biosensors with good stability when stored at room temperature. Enzyme immobilized nanocellulose paper-based sensors can detect human blood glucose concentration for diabetes control at very low price compared to the current commercially available electrode-based glucometers. Further, the diagnostic is only made of paper, which is far more sustainable and easier to dispose of; it represents one of the few non-plastic, non-metal disposable test for developing a more sustainable world. We believe this new concept can be generalized to measure many other bio analytes, each using different enzymes to promote health in remote communities and be integrated with Digital Medicine.

## 2 Methodology

### 2.1 Materials

Bleached *Eucalyptus* Kraft (BEK) pulp, 2,2,6,6-Tetramethylpiperidine-1-oxyl (TEMPO), Sodium Bromide (NaBr) and 12 w/v% Sodium Hypochlorite (NaClO). Hydrochloric acid (HCl), Sodium Hydroxide (NaOH) and Sodium Chloride (NaCl) were diluted for solutions as required. Glucose oxidase and phenol red were purchased from Sigma Aldrich. D(+)-glucose powder was sourced from Merck. Human blood was collected from Australian Red Cross. Glucose colorimetric kit was purchased from Invitrogen.

### 2.2 TEMPO mediated oxidation

The TEMPO-mediated oxidation process producing a low surface charged fibers is based on a previously developed method (Saito et al., 2007). 100 g BEK pulp is suspended in 2500 ml water containing 0.4 g TEMPO and 2.5 g NaBr. The 12 w/v% NaClO solution is initially adjusted to pH 10 *via* addition of 36 w/v% HCl. 100 ml NaClO is added dropwise to the suspension while stirring. The pH of the reaction is maintained at 10 by adding 0.5 M NaOH. The oxidation process is deemed complete when the pH change is negligible. The oxidized fibers are recovered by filtration and stored at 4°C. The TEMPO-oxidized pulp is then dispersed in deionized water at the desired concentration. Fibrillation is accomplished through a high-pressure homogenizer at 1000 bar with two passes. The hydrogel produced by this procedure is stored at 4°C.

### 2.3 Preparation of nanocellulose sensor

5 g of Nanocellulose hydrogel was mixed with 214.77 µl of 372.5 µM GOx and 100 µl of 0.75% phenol red and mixed with magnetic stirrer for an hour. After 1 h, the mixed hydrogel was transferred on a petri dish. The petri dish was previously wrapped with glad wrap to avoid the hydrogel stick to the petri dish surface after drying. Another petri dish wrapped with glad wrap was placed on the top of mixed hydrogel petri dish. Then a fixed weight of glass petri dish was placed on top to press the hydrogel and make a thin layer. Then this setup containing mixed hydrogel was placed into −80°C freezer for at least 4 h followed by freeze drying in a Christ Alpha 2–4 LD Plus freeze dryer for 24 h. The dried samples were cut into 8 mm circles by Epilog Laser cutter with a speed of 100% and 3% power.

### 2.4 Blood glucose level determination

The glucose concentration in the blood sample received from the Australian Red Cross was measured by glucometer (ACCU-CHEK Performa). The glucose concentrations of some of the blood samples were measured by Invitrogen Glucose colorimetric detection kit. This kit comes with glucose standard solution (320 mg/dl), assay buffer (contains detergent and stabilizers), substrate, horseradish peroxidase (HRP) concentrate, glucose oxidase (GoX) concentrate. 1X GoX and 1X HRP was prepared according to the instructions provided by the supplier. The human blood serum was diluted at 1:20 into the assay buffer and 20 µl of plasma was added with 25 µl 1X HRP, 25 µl substrate and 25 µl GoX in 96-half area well plate. The colorimetric reaction was analyzed in a Tecan Infinite M Nano plate reader at 560 nm after 30 min of incubation. The standard curve for glucose was generated by diluting the glucose standard for glucose concentration ranging from 0–32 mg/dl and quantifying the absorbance at 560 nm. The glucose concentrations of unknown samples were read from the standard curve and multiplied by the dilution factor. A few blood samples glucose concentrations were also measured by Melbourne pathology to confirm the accuracy of glucometer.

### 2.5 Spiking glucose concentration in blood plasma

The whole blood samples glucose concentrations were measured by glucometer and then the blood plasma was separated from red blood cells by centrifugation at 4500 rpm for 20 min. The blood plasma was mixed with glucose solution to have the desired concentration for the blood plasma samples. All the samples were mixed with the same volume of glucose solution to maintain consistency in blood plasma dilution.

### 2.6 Sensor testing with different temperatures and glucose concentrations

Dried nanocellulose biosensor sized 8 mm was placed inside a light box and tested for five glucose concentrations: 7, 8, 10, 11, and 13 mM at three different temperatures; cold: 4°C, room: 20°C and hot: 40°C. 10 µl spiked blood plasma was added on the nanocellulose sensor by a 10 µl pipette. Continuous video capturing was done at 4K 60 FPS (frames per second) from the top of a light box (16 × 16” dimmable 70 LED light box) by an iPhone from initial to 25 min. The light box was used to maintain the consistent light intensity throughout the experiments. 4°C testing was done inside a cool room maintained at 4°C and for 40°C temperature, a hot plate was used. Each test was done for four replicates. The images were extracted from the video file for analyzing at different time intervals.

### 2.7 Shelf life testing

Dried nanocellulose biosensor was stored at 23°C and 50% RH for 4 weeks and tested for different glucose concentrations and room temperature as described in Section 2.6.

### 2.8 Image analysis

The images at specific times (30 s, 1, 2, 4, 7, and 10 min) were extracted from the captured videos as image file. The background of these images was removed in Photoshop. The image compilation was done in Adobe Photoshop. The color hue of the images was also quantified in Photoshop.

## 3 Results

A new concept of point of care (POC) paper-sensor using nanocellulose gel/foam was developed to directly measure and rapidly report by colorimetry the glucose concentration in human blood. The variables tested include time, glucose concentration and temperature; this was to evaluate the glucose diagnostic potential under relevant POC conditions. Glucose oxidase immobilized in nanocellulose gel/foam reacts with the blood glucose to produce gluconic acid which decreases pH, change detected and visualized by colorimetry. The effect of contact time was studied to optimize the color development reporting glucose concentration; it was also used to determine how long the reporting color remains stable. The diagnostic was tested at three critical temperatures to report the dependency of reaction rate and color intensity with temperature. Diagnostic performance at room temperature (20°C), sample stored in the fridge (4°C) and during a hot Australian day (40°C) was tested.

### 3.1 Glucose assay

The colorimetric glucose assay developed was validated with a glucose colorimetric kit and by spectrophotometry. Both tests show similar results (Table 1). This confirms the sensitivity and accuracy of glucometer (ACCU-CHEK Performa; purchased from local Chemist Warehouse) reading. Spectrophotometry tests to determine glucose concentrations was only performed for a few samples. This served to calibrate and validate reliable glucometer reading and that the spiked plasma samples have the correct glucose concentration. According to the supplier, the glucometer has standard deviation of ±1.5.

**TABLE 1:** Comparison of glucose concentration in human blood as determined by spectrophotometry and glucometer.

Sample	Glucometer reading (mM)	Spectrophotometry reading (mM)
1	4.20 ± 1.5	3.48 ± 0.35
2	7.00 ± 1.5	7.84 ± 0.63
3	9.98 ± 1.5	9.61 ± 0.27
4	10.00 ± 1.5	10.09 ± 0.72
5	13.00 ± 1.5	12.77 ± 0.99

### 3.2 Effect of time

**FIGURE 2:**
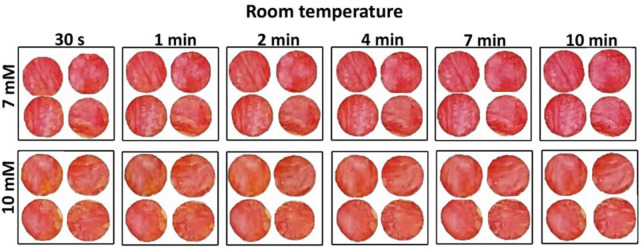
Effect of time on nanocellulose sensor colorimetric measurement performed at room temperature (20°C ± 1°C) for two different glucose concentrations: 7 and 10 mM.

Testing time, defined by the time of blood-enzyme contact, was first optimized to ensure reproducible and sensitive results. This was performed at two temperatures: 20°C (room) and 4°C (fridge). Figure 2 shows the effect of reaction time ranging from 30 s to 10 min for the nanocellulose sensor color at room temperature (20°C). Room temperature images show a more stable color development than at cold temperature (Supplementary Figure S1 in supp info). For cold temperature, both 7 and 10 mM color change over time: 7 mM gets reddish while the 10 mM turns orangish with time. A 7 min blood-sensor contact time was determined as optimum for both room and cold temperature reading. This contact time allows the blood plasma to be fully distributed and to generate a stable and measurable color change using nanocellulose gel/foam.

### 3.3 Effect of temperature

**FIGURE 3:**
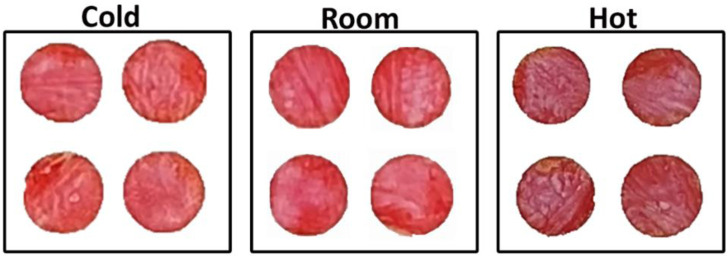
Effect of temperature on nanocellulose biosensor color development and intensity for measurement under cold (4°C ± 1°C), room (20°C ± 1°C) and hot conditions (40°C ± 1°C) after 7 min for 7 mM glucose concentration.

Temperature can be an important parameter for developing colorimetric sensor based on an enzymatic reaction. Testing was conducted at 4°C, 20°C, and 40°C. High temperature (40°C) results in a maroon color for 7 mM glucose concentration in blood plasma, while the lower temperatures develop a red color (Figure 3). There is a slight difference between cold and room; room temperature shows a slightly darker red than cold temperature.

### 3.4 Effect of glucose concentration

**FIGURE 4:**
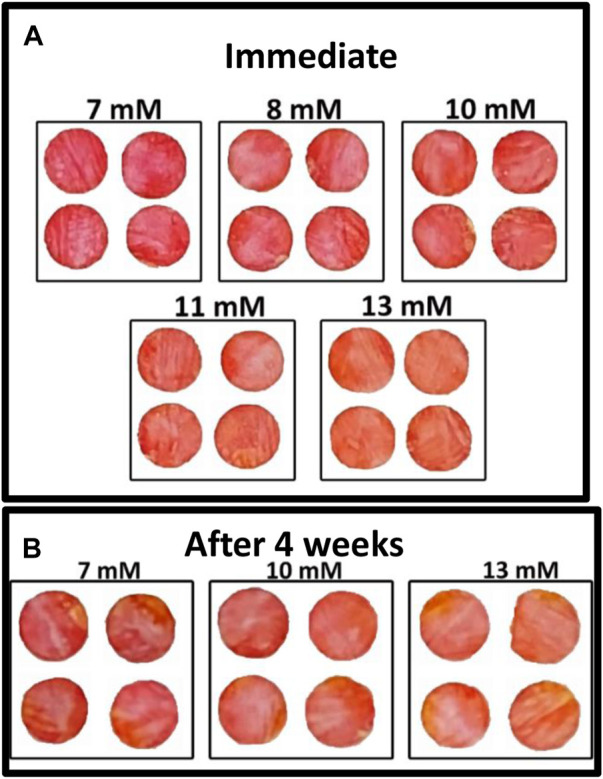
Effect of glucose concentration on colorimetric properties of nanocellulose sensor after 7 mins (room temperature): (**A**) Immediate test and (**B**) Test after 4 weeks aging of the enzyme sensor.

A colorimetric glucose sensor must show visible differences in color in the concentration range of interest. The nanocellulose sensor shows a gradual color change from red to orange as the glucose concentration in blood plasma increases from 7 to 13 mM (medical importance) (Figure 4A). Higher glucose concentrations produce higher concentrations of gluconic acid as catalyzed by glucose oxidase which decreases pH, resulting in a color change, phenol red changes from red to orange as pH drops.

Quantification of biosensor shelf life is important as long-term storage is required of biosensors for economic viability. The nanocellulose biosensor was tested after 4 weeks aging at room temperature in an airtight container (23°C and 50% relative humidity) to quantify its effectivity. Figure 4B shows the gradual color change for different glucose concentrations ranging from 7 to 13 mM. This color change confirms the activity of glucose oxidase after 4 weeks storage.

### 3.5 Effect of fresh blood

**FIGURE 5:**
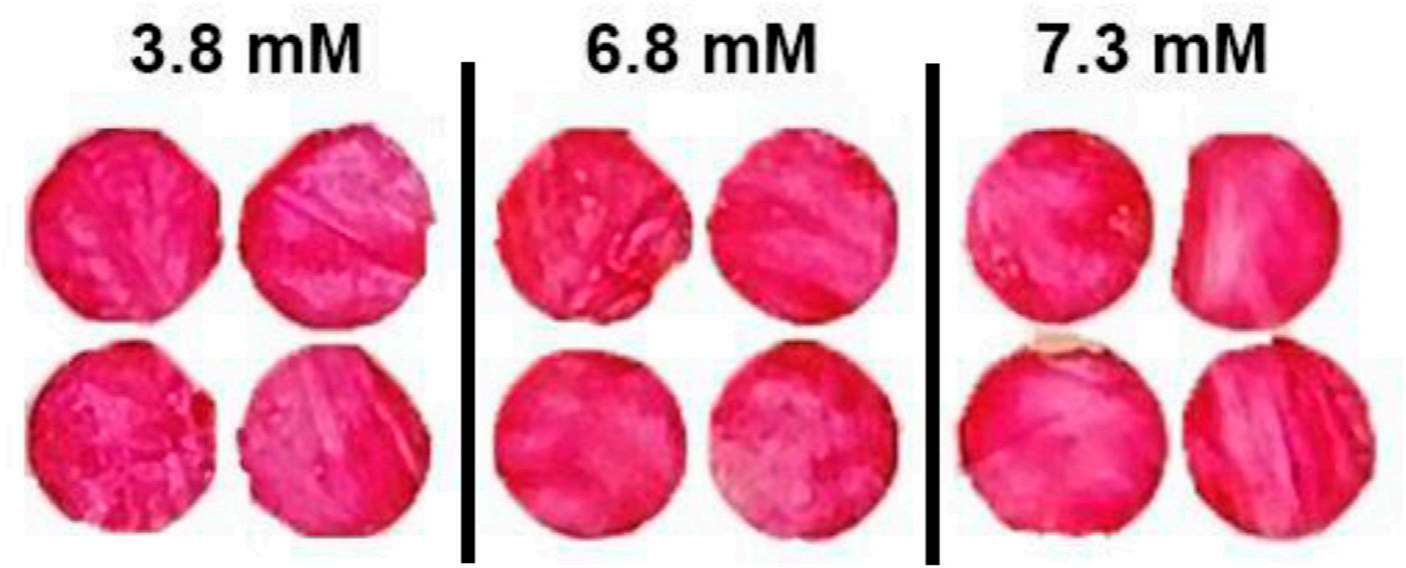
Effect of fresh blood on the colorimetric properties for nanocellulose sensor for reading after 7 min for glucose concentrations of 3.8 to 7.3 mM.

The blood sample from the Australian Red Cross was already 3–4 days old when tested. Hence, colorimetric testing was also performed for fresh blood sample tested within 3 h of blood withdrawn. Figure 5 shows the nanocellulose sensor response for low glucose concentrations for fresh blood samples. All the blood samples (non-spiked) tested had a glucose concentration lower than 8 mM, below the sensor’s sensitivity. However, a more pinkish color was observed on the sensor for fresh blood sample compared to the old blood samples (Figure 4).

### 3.6 Designing a diagnostic prototype

**FIGURE 6:**
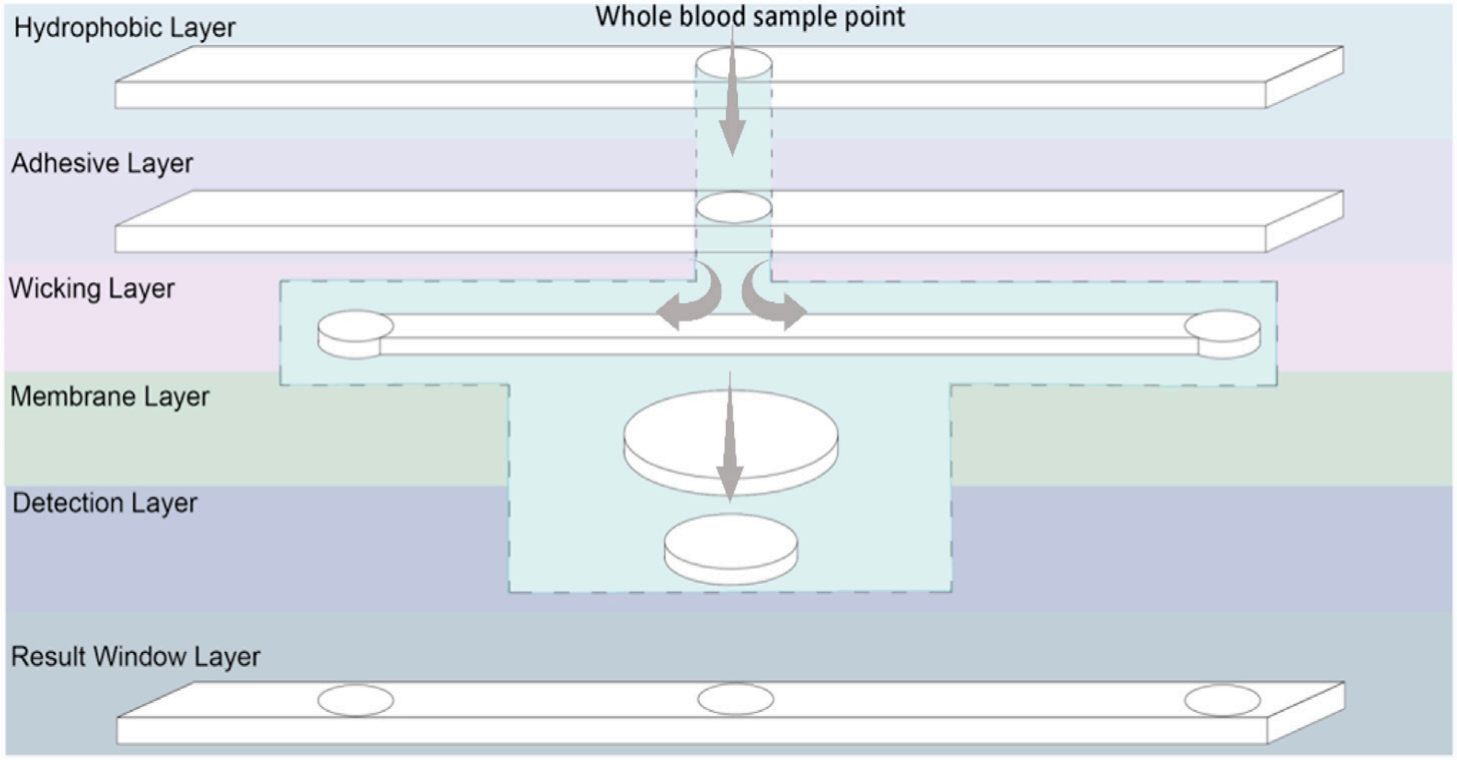
Schematic representation of a laminated glucose diagnostic representing the Microfluidic 3D pathway of the handheld device.

A 3D multilayer laminate based diagnostic device was designed. The device has three functions to achieve. First, to measure and display testing reading time; second, to remove RBC, as only plasma is analysed for glucose content; and third, to measure and colorimetrically report glucose concentration. The composition and 3D pathway of the device layers is presented in Figure 6. This device consists of six layers: 1) a hydrophobic layer on top to prevent leakages, 2) an adhesive layer to keep the layers together and control spacing, 3) a wicking layer to monitor reaction time for optimum reading, 4) a membrane layer to separate red blood cells from plasma, 5) the colorimetric detection layer (sensor part), and 6) a hydrophobic result window layer. Three microfluidics are combined: a time-controlled wicking channel (layer 3), a paper-based plasma separating membrane (layer 4), and a detection zone (layer 5). However, the time-controlled wicking channel can be removed if the users can monitor their reaction time.

The time-controlled wicking channel is such as the blood reaches the left corner of the channel at the optimized time for the glucose concentration to be recorded and displayed. The right side of the wicking channel is longer than the left side which requires more time to transport the blood on the right edge indicating the color reading is expired. The wicking speed in the wicking channel can be controlled by the porosity and thickness of the paper used. Tissue paper from Kimberly Clark Experimental Forming Unit (EFU), Neenah (WI), United States was selected (Hertaeg et al., 2021a; Hertaeg et al., 2021b); it is available at a range of thickness. The paper-based RBC separator prevents the need of centrifugation for blood preparation; the user applies blood directly to the sample area. The membrane relies on the paper pores structure to filtrate the (7–8 µm (Kinnunen et al., 2011)) interfering pigmented red blood cells; leaving a transparent yellow tinted plasma solution to react within the detection zone (PALL, 2021). When a blood sample is deposited onto the sampling area, it wicks both laterally and vertically through the channel. From there, the blood sample travels to the membrane layer vertically which separates red blood cells and only let the plasma pass through the membrane to the next layer, which is the detection zone. A hydrophobic waxed circle in the centre of the membrane prevents the reddening membrane from altering the visual reading of the nanocellulose superabsorbent zone. The nanocellulose superabsorbent in the detection layer is adhered to the membrane to prevent bypassing; the membrane undergoes a waxing process that hydrophobically blocks the fluid from reaching the membrane edges and contaminate the adjacent layers. The blood plasma reacts with enzyme and colorimetry reagents within the nanocellulose superabsorbent resulting in a color change, visible through the middle result window. Once the blood has wicked to the left end of the lateral wicking channel, it is seen through the result window, indicating that the color can now be read. The membrane used in this design to separate RBC was not efficient enough to produce clear plasma solution and pass the required amount of plasma to the detection zone. This membrane needs to be engineered to have a porosity smaller than RBC with faster separation efficiency.

## 4 Discussion

### 4.1 Governing variables

We developed a nanocellulose based colorimetric sensor by engineering its unique high porosity and high surface area properties. This paper-based sensor is tested for different glucose concentration, reaction time, reaction temperature and stability over 4 weeks’ time. The sensor successfully shows color change at different glucose concentrations, confirming its functionality at the blood glucose critical to diabetes monitoring.

Glucose oxidase immobilized in nanocellulose oxidizes glucose into D-glucono-1,5 lactone, which then hydrolyses into gluconic acid. H_2_O_2_ is produced as a by-product in this reaction and O_2_ (from air) is a reactant (Kornecki et al., 2020). This acidic medium changes the pH of the system which is indicated by pH sensitive phenol red color change in the sensor. The reaction visually proceeds with time (Figure 2). The reaction is quicker at higher temperature with darkness increasing with temperature (Figure 3), revealing the higher catalysis reaction rate of glucose by glucose oxidase at higher temperature. This is consistent with literature reporting reaction rate of glucose oxidase at temperature of 25°C–60°C. The reaction rate increases with temperature following a second order reaction rate constant (Odebunmi and Owalude, 2007). The optimized time to read the sensor color can differ for large temperature differences.

**FIGURE 7:**
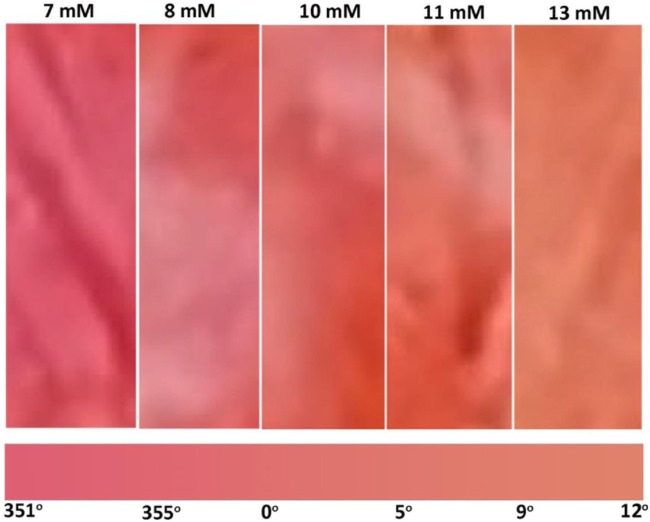
Gradient of color change produced by glucose concentration of medical interest ranging from 7 to 13 mM.

The sensor developed quantifies the difference in glucose concentration in blood plasma by reporting the color changing from red to orange (Figure 4). Normal blood glucose concentrations for non-diabetics are under 7.8 mM 2 h after a meal. Glucose levels in between 7.8 and 11 mM indicate prediabetes, and higher than 11 mM is diabetes. Our glucose sensor can consistently determine the difference between 7 and 8–10 mM (Figure 4) which determines the occurrence of diabetes. The color difference between 10, 11, and 13 mM also differentiates the state between pre-diabetes and diabetes. The sensor color change is shown as a color gradient in Figure 7. This is confirmed with color hue calculation as shown in the image by the horizontal gradient bar. Summary of color theory and the reasons to choose color hue is given in supplementary information (Supplementary Figures S2, S3). Color hue of the images changes from 351° to 12°, indicating quantitative confirmation of color change. This number can be integrated with any device to show directly a glucose concentration number instead of comparing the color with a standard provided. However, this sensor can work independently without incorporating into an electronic device.

This predictable and reproducible color change of enzyme immobilized pH based nanocellulose sensor enables the design of diagnostic prototypes. The conceptual device is designed for simplicity to be used by low-income users in remoteness and having restricted electricity. The three step testing procedure follows the ASSURED principles—affordable, specific, sensitive, user-friendly, rapid and robust, equipment free, and deliverable (Byrnes et al., 2013).

### 4.2 Diagnostic device concept

**FIGURE 8:**
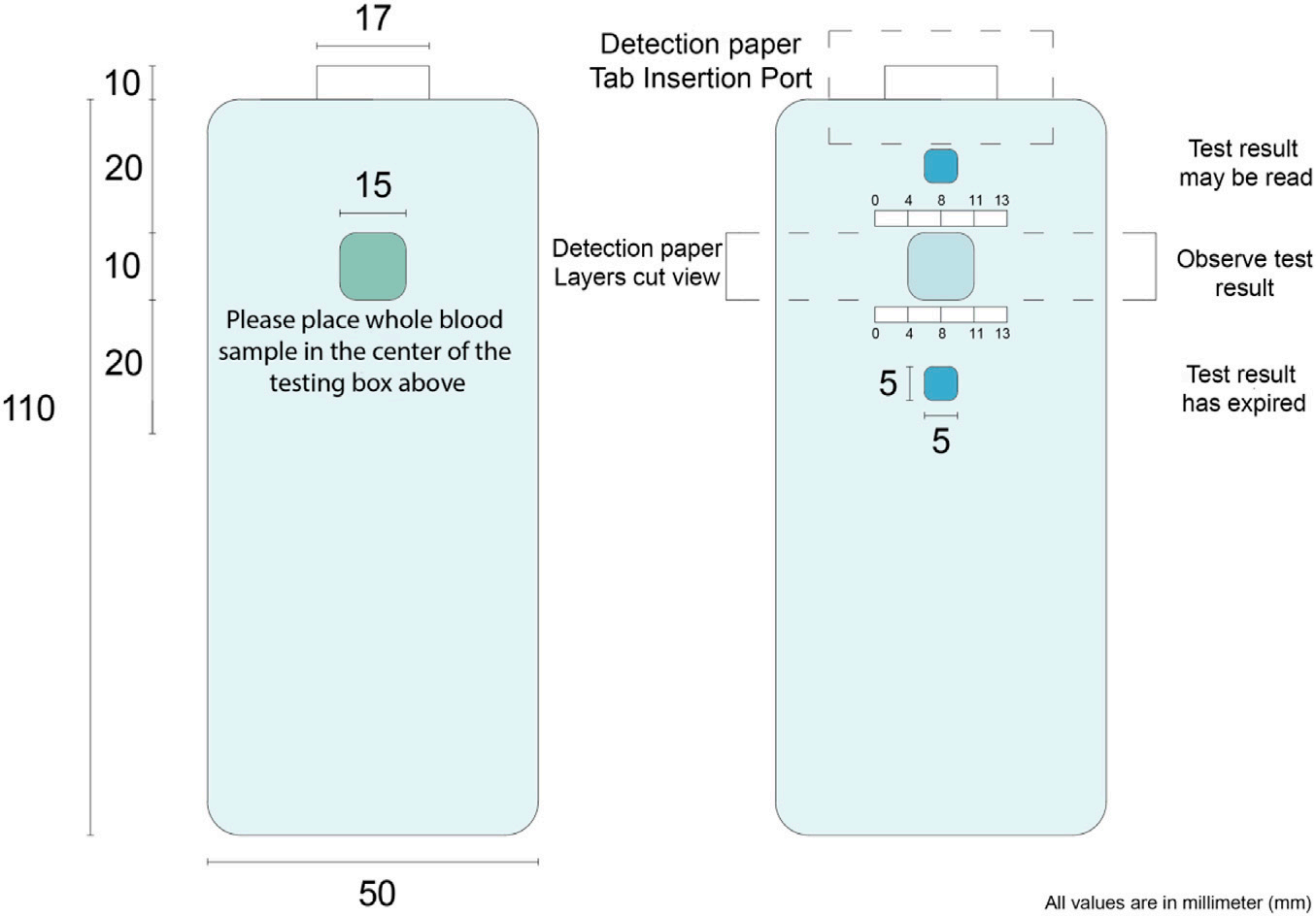
Nanocellulose sensor device concept. The numbers on the image represent the distance in mm. These are designed for user friendly device.

Figure 8 shows the device design based on a nanocellulose sensor. A single-use diagnostic strip (Figure 6) is inserted within the user’s reusable plastic casing that details the three-step procedure. A sample of approximately 20 µl of blood–obtained by pricking a finger with a lancet—is dropped onto the whole blood detection zone. Turning the device over, an indicator box at the top will turn red due to blood wicking after a certain time, communicating the nanocellulose diagnostic zone is available to be read for results. The standard colors at different glucose concentrations is given on the device. Once the bottom indicator box turns red after another specific time, the results have expired. Encouraging a new generation of environmentally conscious single-use diagnostic device—once testing concludes the biodegradable paper strip may be disposed of with the individual’s plastic insertion device kept for reuse.

## 5 Conclusion

This study investigates nanocellulose gel as a generic medium for the colorimetric detection of glucose in human blood based on an enzyme catalyzed reaction of glucose. A novel handheld paper-based glucose POC diagnostic device is engineered to enable vulnerable communities to self-monitor their glucose level continuously. This colorimetric method is assessed in terms of temperature, time and glucose concentrations to develop a robust diagnostic. The color change is determined both qualitatively and quantitively to confirm accuracy and consistency. The sensor aims to determine the onset of diabetes; it is not designed to measure the glucose concentration very precisely with a decimal place accuracy. High diabetes is easy to detect; however, here, we are targeting individuals who have not yet been diagnosed with diabetes.

Phenol red-based glucose oxidized immobilized nanocellulose sensor differentiates glucose concentrations in blood plasma by showing a gradual color change from red to orange. It is sensitive in the 7–13 mM concentration range of interest for diabetes detection. The relation between color change and reaction time is quantified and optimized to provide a stable and accurate color. The immobilized enzyme remains active up to 4 weeks at room temperature, providing a minimal diagnostic shelf life. This contrasts to the usual 3 weeks shelf life at 4°C for enzymes in solution. This colorimetric method shows high temperature sensitivity at very high temperatures (40°C). However, the sensor developed is stable at the usual test temperature, typically ranging from 20°C to 30°C. This research defines the potential of paper-based enzyme colorimetry glucose device and highlights expanded application of nanocellulose superabsorbent diagnostic devices using a wide range of enzyme to detect a board array of diseases.

## Data Availability

The original contributions presented in the study are included in the article/Supplementary Material, further inquiries can be directed to the corresponding author.
