# Supplementary material for: Nanocellulose coated paper diagnostic to measure glucose concentration in human blood
Source: Front Bioeng Biotechnol. 2022 Nov 22;10:1052242. doi: 10.3389/fbioe.2022.1052242 (PMC9723229; doi:10.3389/fbioe.2022.1052242)
Supplement: Supplementary file 1 [file DataSheet1.docx]

**Nanocellulose coated paper diagnostic to measure glucose concentration in human blood**

**Supplementary Information**


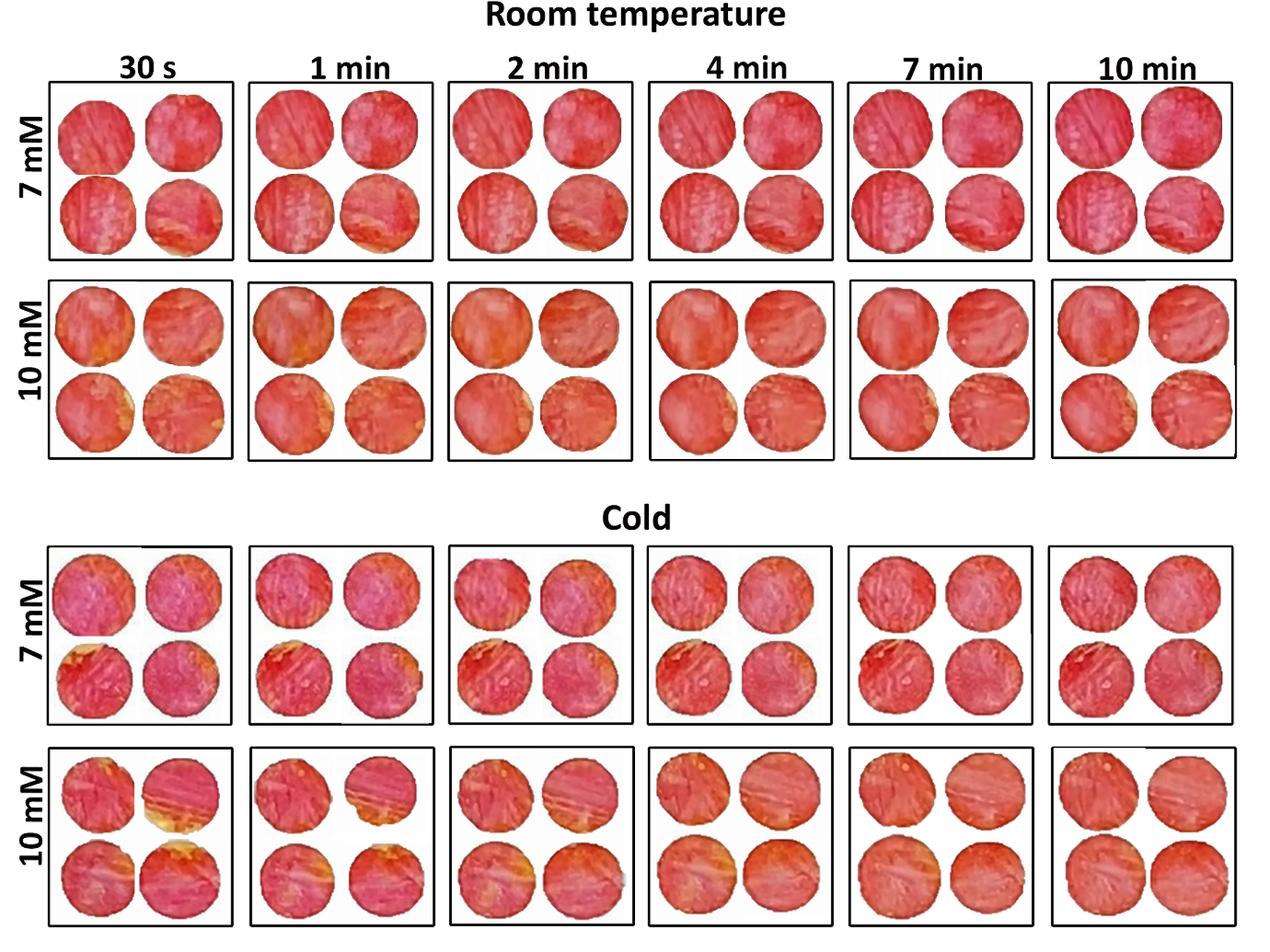


**Figure S1:** Comparison of color change at a function of reaction time for room and cold temperature.

**Color Analysis**

There are three primary ways to describe a color: hue, chroma and value. Hue is the dimension of color we instantly experience when we look at the color. Hue distinguishes from one color to another, such as, red, orange, yellow, blue, violet and green. Hue is illustrated by a thermometer in Figure S2 as it describes the color temperature. Value, also called lightness or luminosity, measures the lightness or darkness of a color. Value is described as a circle in the figure S2. Value defines a color on a basis of how close the color is in regards to white or black. Chroma, also called saturation, indicates the degree of intensity or purity. Chroma is indicated by sun in the Figure S2 as it indicates the degree of clarity/ saturation/intensity.


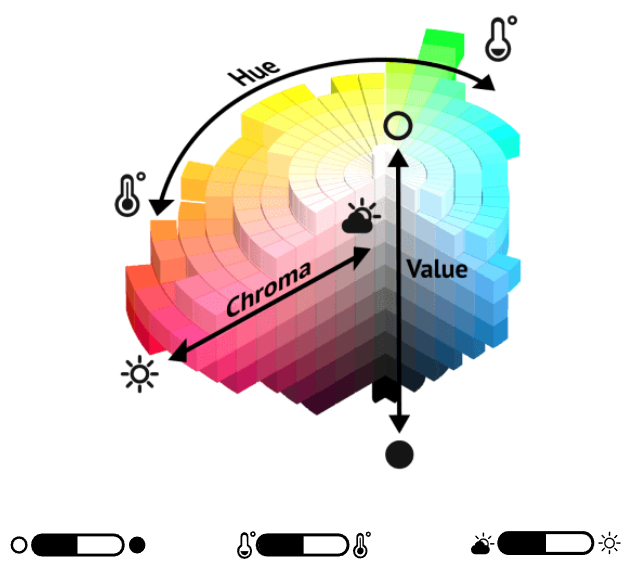


Figure S2: Color scale showing hue, chroma and value [1].

From the above discussion, it is evident that color hue is the best option to describe the color difference as it distinguishes different colors. So, color hue is chosen to distinguish between different samples colors for our glucose sensor described in Figure 7. Figure S3 presents the hue wheel indicating different numbers for different colors for a range of 0 to 360^o^.


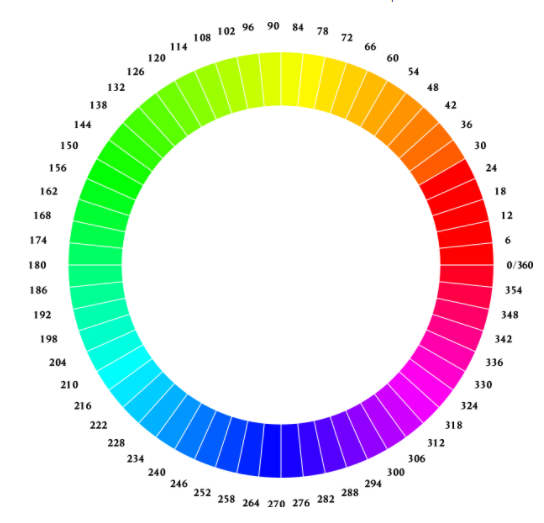


Figure S3: Color wheel indicating different hue colors. The numbers on the wheel are in degrees [2].

**Reference**

1. Color & Style. *Compatibility with Color Systems and Names*. Available from: <https://en.color-style.com/compatibility/>.

2. Pinterest. *Chromatic Wheel 1*. [cited 2021; Available from: <https://www.pinterest.at/pin/114349278015552260/?amp_client_id=CLIENT_ID(_)&mweb_unauth_id=&simplified=true>.
